# Protecting endangered species in the USA requires both public and private land conservation

**DOI:** 10.1038/s41598-020-68780-y

**Published:** 2020-07-17

**Authors:** Niall G. Clancy, John P. Draper, J. Marshall Wolf, Umarfarooq A. Abdulwahab, Maya C. Pendleton, Soren Brothers, Janice Brahney, Jennifer Weathered, Edd Hammill, Trisha B. Atwood

**Affiliations:** 1 Department of Watershed Sciences and the Ecology Center, Utah State University, Logan, UT USA; 2 Montana Fish, Wildlife and Parks, Kalispell, MT USA

**Keywords:** Ecology, Conservation biology

## Abstract

Crucial to the successful conservation of endangered species is the overlap of their ranges with protected areas. We analyzed protected areas in the continental USA to assess the extent to which they covered the ranges of endangered tetrapods. We show that in 80% of ecoregions, protected areas offer equal (25%) or worse (55%) protection for species than if their locations were chosen at random. Additionally, we demonstrate that it is possible to achieve sufficient protection for 100% of the USA’s endangered tetrapods through targeted protection of undeveloped public and private lands. Our results highlight that the USA is likely to fall short of its commitments to halting biodiversity loss unless more considerable investments in both public and private land conservation are made.

## Introduction

In 2010, 194 countries committed to halting biodiversity loss by adopting the Aichi Biodiversity Targets and Sustainable Development Goals^1^. Crucial to the success of this commitment is the protection of important habitat to support terrestrial and aquatic biodiversity. Highly protected areas (e.g., national monuments, national parks, and wilderness areas) that heavily restrict anthropogenic activities are the current mainstay for biodiversity conservation because, in general, well managed and effectively placed protected areas have been shown to increase species richness and abundance relative to unprotected areas^2–5^. As a result, the spatial extent of protected areas is used to monitor global progress towards achieving Aichi Biodiversity Targets and Sustainable Development Goals, and Aichi Target 11 specifically identified that 17% of terrestrial areas and inland waters needed to be protected by 2020^1^. Unfortunately, as 2019 came to a close only 15.2% of global land was located within protected areas^6^. In 2020, the Convention on Biological Diversity (CBD) will adopt a new global biodiversity framework for the post-2020 era with new targets set for 2050. To develop this new framework, the CBD will need to review its successes and failures with the previous framework.

One of the major criticisms of Aichi Target 11 is that it is an area-based target that can be met with little relevance to the protection of biodiversity^7^. Although the designation of a protected area counts towards Aichi Target 11, protected areas do not have to be designated with the primary goal of protecting biodiversity. This is especially true in countries such as the United States of America (USA) where protected areas have historically been designated for reasons other than biodiversity, such as cultural and historical significance^8^, or lack of agricultural value^9^. As a result, recent analyses have brought into question whether existing protected areas are actually improving the conservation status of imperiled species^3, 4^, and whether they should effectively be counted towards reaching Aichi Target 11. To determine the extent to which current protected areas are aiding in the protection of imperiled species and how much additional land is required to protect species, we must understand the overlap between protected areas and species ranges.

Given that the ultimate goal of achieving Aichi Target 11 is to protect biodiversity and the services it provides, new lands added to reach current and future targets should be designated with the specific goal of protecting biodiversity, especially threatened and endangered species. Currently, only 7.1% of the USA’s land is in a highly protected status that is managed to preserve biodiversity^8,10^. The most common pathway by which the government designates new lands as protected is through the conversion of existing public land to a protected status [e.g., the conversion of Bureau of Land Management (BLM) land to a National Monument or Wilderness Area]^11^. However, the political appetite for the conversion of public lands to highly protected areas is not always favorable^12^. Furthermore, the availability of public lands for conversion to protected areas may be limited. For example, ~ 95% of the land in the state of Texas, USA, is privately owned. These political and logistical obstacles may mean that private lands such as conservation easements may need to take a more prominent role in meeting conservation targets in the USA^13,14^.

Our study aimed to assess how current protected areas in the continental USA are contributing to the protection of its most imperiled species, and how the conversion of existing public and private lands to a highly protected status can aid the USA further in safeguarding those species. We used a null modeling approach^15,16^ to analyze whether current protected areas include threatened and endangered species and sub-species (hereon referred to as ‘endangered species’) better than if they had been placed at random. We then assessed how many endangered species had 30% of their range inside the current layout of protected areas. In the absence of species-specific population viability analysis, a rule of thumb suggests that 30% of a species’ range must be protected for it to persist in the wild^17^. We then further analyzed whether or not the capacity to protect more endangered species exists through the targeted conversion of undeveloped public and private lands to a highly protected status. For all of our assessments, we use an ecoregion-based approach, as ecoregions have been shown to represent the broadest inclusion of diverse habitats and species^18^.

## Results and discussion

**Figure 1:**
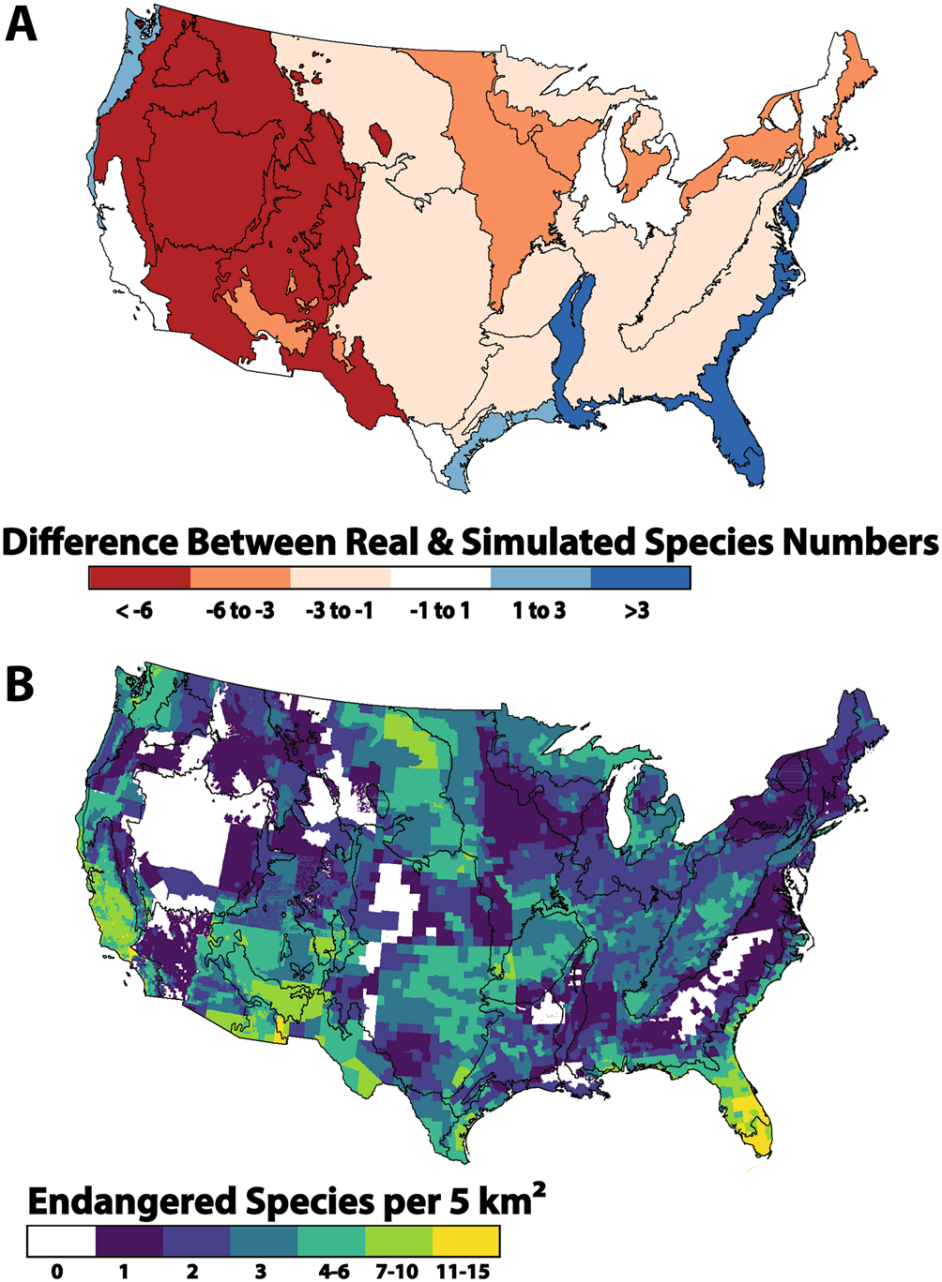
Null modeling results and endangered species richness within the USA. **(a)** Difference between the current number of endangered species with any part of their range inside highly protected areas and the average results of 1,000 random placements of those highly protected areas. Warm colors show regions that performed worse than a random placement, while cool colors indicate regions that performed better than random. Ecoregion data were obtained from The Nature Conservancy^34^. **(b)** Number of endangered tetrapod species per 5 km^2^ pixel, species distribution data were obtained from The USFWS Environmental Conservation Online System^47^.

While biodiversity conservation is cited as a priority for many existing highly protected areas, our null modeling results indicate that the placement of protected areas in the USA has largely failed to include at-risk species. For a large number of ecoregions, especially in the western states, we found that endangered species currently have less of their range contained within protected areas than if these areas had been placed at random (Fig. 1a). Across the entire continental USA, we found that highly protected areas in 55% of ecoregions were worse at protecting the ranges of endangered species than if their location had been chosen at random within the same ecoregion (Fig. 1a). An additional 25% of ecoregions performed no better than random. This lack of coverage for at-risk species likely stems from the motivations for initial placement, as many protected areas were placed based on scenic beauty^8^ or poor agricultural potential^9^. The remaining 20% of ecoregions that performed better than random were coastal regions with moderate to high numbers of endangered species (Fig. 1a, b). Since coastal areas tend to have high human population densities^19^, coastal protected areas may be performing better than random because sensitive species have already been extirpated outside protected areas, and current species’ ranges now exist primarily in those protected areas.

The USA is not alone in the underperformance of its protected areas in helping to preserve biodiversity. While protected areas globally are associated with higher levels of biodiversity^2^, regional and country-level studies have found that protected areas in Australia, Canada, Laos and parts of the neotropics are also safeguarding endangered species ranges, endemic species, or total species richness worse than if protected areas had been randomly placed^20–26^. The diversity of countries with inadequate biodiversity protection suggests that this problem is not just in developed countries where valuable land may already have been co-opted for human use. These combined results suggest that even if we meet Aichi Target 11 by protecting 17% of global terrestrial areas and inland waters, there may be minimal benefits for endangered species. Thus, the USA and several other countries need to focus new conservation efforts on creating protected areas that are specifically placed to cover the ranges of endangered species to aid their survival.

**Figure 2:**
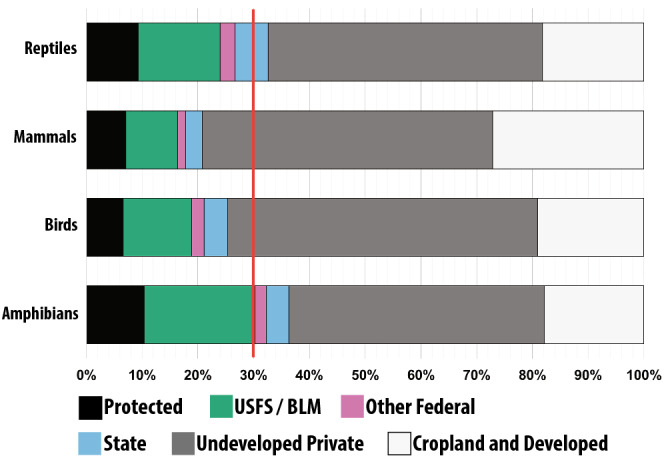
The average percent of endangered species ranges within each type of land designation by tetrapod class. The red line shows the 30% threshold for adequate protection of a species.

While the effectiveness of protected areas for preserving biodiversity integrates the geographical location, habitat quality, and human use of the protected area^20,27^, achieving biodiversity targets requires sufficient overlap between protected areas and the species of interest. We found that the creation of additional highly protected areas through the conversion of existing public lands to highly protected areas would increase the number of at-risk species that have 30% of their range protected. However, this conversion would not be sufficient to protect 30% of the range of a majority of the USA’s endangered species (Supplementary Table S1; Fig. 2) and is also unlikely to be politically feasible given the multiple use mandate of public lands in the USA^11^.

**Figure 3:**
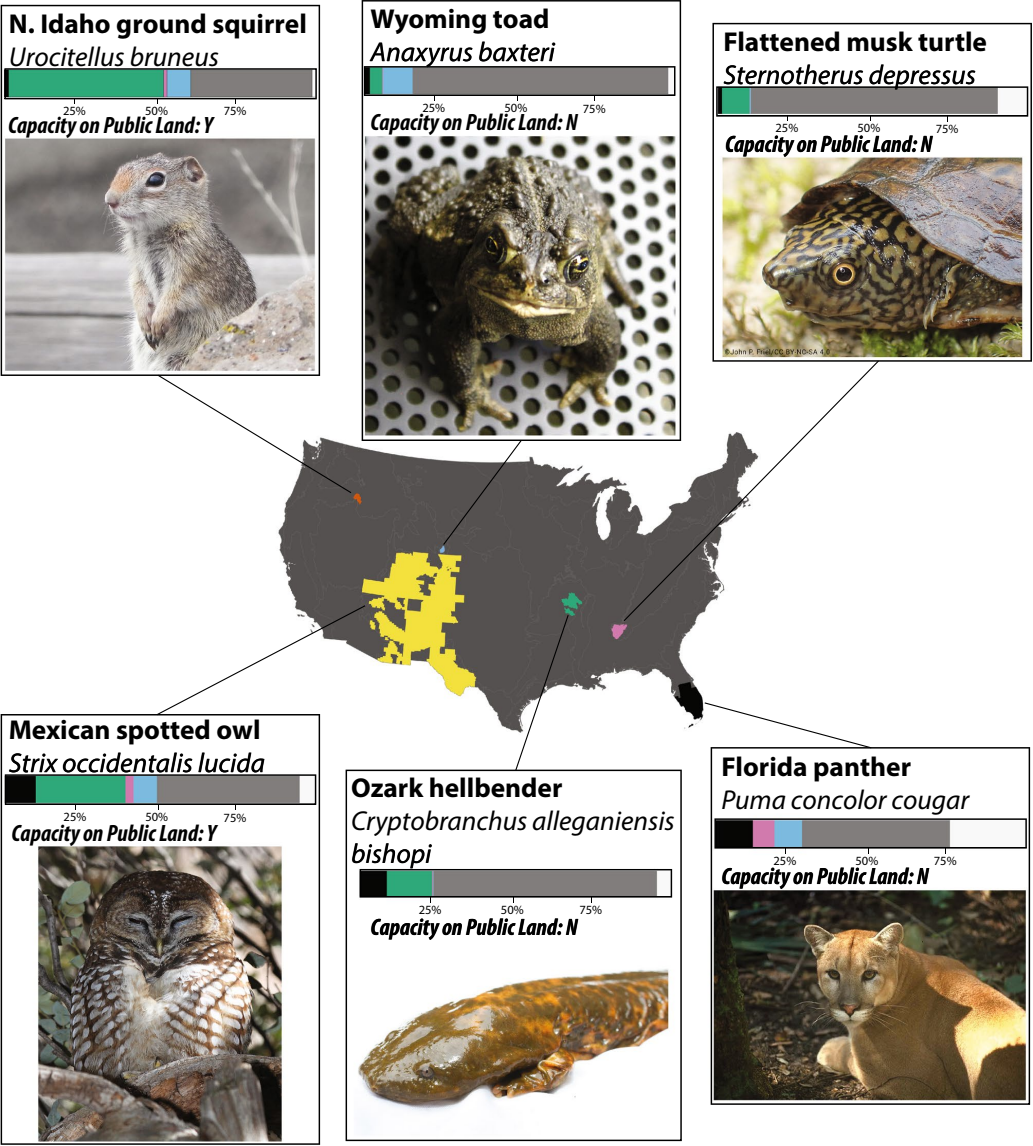
Examples of endangered species that would benefit from different combinations of public and private land conservation. Map colors show species’ ranges; *Urocitellus bruneus:* orange*, Anaxyrus baxteri*: blue, *Sternotherus depressus*: pink, *Strix occidentalis lucida:* yellow, *Cryptobranchus alleganiensis bishop*: green, *Puma concolor cougar*: black. Bar graphs indicate the percent of species ranges within each type of land designation; black: currently protected federal land, green: U.S. Forest Service and Bureau of Land Management, pink: other federal lands (e.g., Bureau of Reclamation, Department of Defense, etc.); blue: state land; grey: undeveloped private land; white: private cropland and developed land. Landcover data obtained from Bureau Land Management GIS repository^51^, species distributions were obtained from the USFWS Environmental Conservation Online System^47^. Capacity on Public Land indicates that at least 30% of the given species’ range is within federal or state lands (Y), or if less than 30% of the given species range is within federal or state lands (N). Photo credits clockwise from top-left: USFS Region 4; Ryan Moehring/USWFS; John P. Friel; Connie Bransilver; Brian Gratwicke; and Gary L. Clark.

At present, only 21 (13%) endangered species meet the minimum threshold of having 30% of their range within protected areas (Supplementary Table S1). If private lands managed specifically for conservation are added to this analysis, we found that the number of species with > 30% of their range protected does not increase (Supplementary Table S1). This lack of additional species receiving adequate protection when private conservation easements are added is not surprising, as these areas are collectively about 24% of the size of highly protected areas. We found that the USA could protect a total of 59 (37%) of its endangered species by conferring highly protected status to United States Forest Service (USFS) and BLM lands (Supplementary Table S1; Fig. 2). Protection of additional federal (e.g., Bureau of Reclamation, Department of Defense lands, etc.) and state lands would raise this number to 68 (43%) endangered species (Supplementary Table S1; Fig. 2). One example of a species that can be adequately protected on federal and state land is the Northern Idaho ground squirrel (*Urocitellus brunneus*). Currently, only 1.9% of *U. brunneus’* range falls within protected areas, but an additional 51.4% of its range could be protected if other federal lands, such as portions of the Payette National Forest, were highly protected (Fig. 3). Other species for which adequate protection is achievable on public lands include the Mexican spotted owl (*Strix occidentalis lucid*a) and Virginia big-eared bat (*Corynorhinus townsendii virginianus*); (Fig. 3; Supplementary Table S1).

One of the most important findings from our analyses is that the USA has not lost its capacity to protect 100% of its endangered tetrapods adequately. Using the National Land Cover Database^28^, we found that all of the remaining 91 endangered species (57%) can be adequately protected through increased conservation on a combination of undeveloped public and private lands (Figs. 2, 3; Supplementary Table S1). Example species that would benefit from public and private land conversion to a protected status include the Ozark hellbender (*Cryptobranchus alleganiensis bishopi*), flattened musk turtle (*Sternotherus depressus*), Florida panther (*Puma concolor cougar*), and Wyoming toad (*Anaxyrus baxteri*); (Fig. 3). The optimal configuration of a new USA protected area network will require systematic planning because multiple configurations could adequately protect all of the USA’s endangered species. Spatial prioritization will require a framework that takes into account both economic (e.g., cost for protecting different sites, lost opportunities costs^29,30^), and biological (e.g., network connectivity)^14^ factors.

Our analyses suggest that to adequately protect its endangered species, in addition to greater protection of public lands, the USA would need to make considerable investments in private land conservation through efforts such as conservation easements^31^. Reflecting on their growing importance in the field of conservation, a recent review of the private lands literature found that conservation easements were the most frequently addressed form of biodiversity protection on private lands^32^. Crucial to the implementation of these endeavors will be a greater understanding of factors that increase the success of actions on private lands, such as engagement with local stakeholders, public acceptance, and financial incentives^13^.

Despite the great strides made towards meeting Aichi Target 11, our results highlight that protected areas in the USA are failing to sufficiently protect biodiversity because there is poor spatial overlap between endangered species and the placement of current protected areas. While the capacity exists on public lands to double the number of endangered species sufficiently protected (Fig. 2), over half of all endangered tetrapods in the continental USA require conservation on private lands for at least 30% of their range to be protected. Importantly, our analyses indicate that the capacity to meet this 30% threshold for all of the continental USA’s endangered species still exists on undeveloped public and private lands. To truly safeguard biodiversity^33^, we must ensure that new protected areas are not only designated in sufficient quantity, but also in locations suitable to imperiled species. Thus, to adequately protect all of the continental USA’s endangered species, a new protected area network must consider utilizing both public and private lands. In our analysis, we focused on species listed as threatened or endangered; however, this list of species should be considered conservative as there are many vulnerable species with declining populations that have not yet been listed. It is critical that protected lands contributing to the progress of Aichi Target 11 sufficiently protect the world’s most vulnerable species. Failure to do so undermines our true commitments to the Aichi Biodiversity Targets, the Sustainable Development Goals, and our fight against biodiversity loss.

## Methods

### Null modeling

To assess if current highly protected areas are conserving endangered species, we used a null modeling approach^16^ to determine whether highly protected areas within each of the Environmental Protection Agency’s (EPA) level-II ecoregions^34^ contained within their borders more endangered species than if they were placed at random^15^. Ecoregions are designated by the EPA based on ecosystem components such as soil, landform, and major vegetation types^35^, and have been used extensively in conservation planning studies to ensure the creation of networks that represent the broadest range of diverse habitats and species. Protected areas were those designated as either Protected Areas Database of the United States (PAD-US) GAP 1 or 2 (“managed for biodiversity with no extractive uses”^10^). The locations of these protected areas were obtained from the database maintained by the United States Geological Survey (USGS) Gap Analysis Program (GAP) using ESRI ArcGIS 10. Protected areas below GAP 2 were also removed because they are not managed explicitly for biodiversity conservation^36^. All protected areas less than 5 km^2^ were removed from the analysis as we did not want to over-represent the number of endangered species that had part of their range protected. We understand that for many smaller species, having less than 5 km^2^ of their range in a protected area would be sufficient to ensure their survival, however, this would not be the case for many larger species that require large amounts of land. Because our study integrates across many species with varying body sizes and home ranges, we set a conservative cut-off of 5 km^2^. We do not wish to diminish the importance of small protected areas, and understand they are important as refuges^37^ and act as corridors for species with large home ranges^38,39^. In addition, our analyses do not account for the fact that many species have seasonal distributions, requiring them to migrate between different locations using corridors. Our goal was to focus on larger protected areas that have the potential to support populations within their boundaries. Analyses were conducted using packages sp, raster, rgdal, rgeos, tmap, abind, dplyr, and maptools^40–45^ in the statistical programming package R^46^.

Protected areas that spanned two or more ecoregions were split into subordinate parts. Range shapefiles for each endangered tetrapod species within the continental USA were obtained from the U.S. Fish & Wildlife Service’s Environmental Conservation Online System (ECOS) database^47^. The number of endangered species in each ecoregion, whose ranges were located within protected area boundaries, was recorded. To create a random distribution by which the current placement of protected areas would be compared, we used shapefiles of each protected area and then randomly moved their location in both position and orientation within its given ecoregion and re-sampled 1,000 times for endangered species occurrence. We opted to constrain the analysis at the ecoregion scale because ecoregions-based conservation planning has been shown to be effective at protecting species-, community-, and ecosystem-level diversity^18,35,48,49^. Due to the random placement of the protected areas, there is the potential for protected areas to overlap. However, a key assumption of the null modeling approach is that the placement of the protected areas is random, so we did not constrain the placement of the areas. Given that overlapping protected areas would reduce the total area designated as protected in each ecoregion, our analysis of whether existing protected areas are performing better than random should be considered conservative, as overlaps cannot occur in the existing locations, but can in the modeled locations. A count of total unique endangered species was tabulated for each ecoregion for the existing protected area distribution, and for each of the 1,000 iterations. We considered existing protected areas to be performing “better” or “worse” than random only if there was at least a ± 1 species difference between the two.

## Public versus private lands comparison

In order to identify potential alternative methods through which endangered species could receive sufficient protection, we examined the conservation options available on undeveloped public and private lands. We considered a threshold of 30% of a species’ range included in protected areas as adequate protection. While each species will have a minimum population size that is necessary for the species to persist, the 30% threshold was used as it represents a baseline “rule-of-thumb” in the absence of species-specific population viability analyses^17^. Therefore, we sought to determine whether the USA’s highly protected areas are sufficient to cover 30% of each species’ range for the 159 unique populations of endangered tetrapods listed under the USA’s Endangered Species Act^47^. Following calculations of the amount of each species range covered by public protected areas, we then used the National Conservation Easement Database^50^ to determine the number of additional species being protected on private lands managed specifically for conservation (i.e., conservation easements). Finally, we determined how many additional endangered species could be conserved through the targeted conversion of existing public lands to a highly protected status or through the protection of private lands through conservation easements. A common method for creating new highly protected areas in the USA is through the designation of national parks, monuments, or wilderness areas within the boundaries of existing federal and state (hereon, “public”) lands, especially lands managed by the USFS) and BLM^11^. Using ArcGIS 10, USFS and BLM shapefiles were extracted from the BLM National Surface Management Agency GIS^51^ and protected areas, other federal, and state lands from the USGSGAP^35^. We calculated the total area of each species’ range within each land management type by intersecting endangered species’ range shapefiles with the public lands shapefile. Specifically, we looked at the number of endangered species that had > 30% of their ranges on public lands that are not designated as GAP 1 or 2, as these lands could be considered available for conversion to protected areas. We generated a private lands shapefile by merging all the public lands and protected areas shapefiles and subtracting this from the USA shapefile, leaving only private lands. The private lands shapefile was then used to mask the National Land Cover Database^11^ so that the resulting raster contained land cover data only for private lands. The range of each species on undeveloped private lands was iteratively tabulated by overlaying the species’ shapefiles over the private lands only version of National Land Cover Database that we generated. Undeveloped private lands were those not categorized as any level of ‘developed’ or ‘cultivated crops.’ The proportion of each species total range within the continental USA was then calculated by land type.

## Data availability

A list of endangered and threatened terrestrial tetrapods found within the continental USA, and the proportion of their range encompassed by land management type is provided in Supplementary tables 1 & 2.

## Supplementary information

Supplementary Tables
