# Supplementary material for: Protecting endangered species in the USA requires both public and private land conservation
Source: Sci Rep. 2020 Jul 17;10:11925. doi: 10.1038/s41598-020-68780-y (PMC7368077; doi:10.1038/s41598-020-68780-y)
Supplement: Supplementary file 1 — Supplementary Tables [file 41598_2020_68780_MOESM1_ESM.docx]

Supplementary Materials for

Protecting endangered species in the USA requires both public and private land conservation

Niall G. Clancy†, John Draper†, J. Marshall Wolf, Umarfarooq A. Abdulwahab, Maya Pendleton, Soren Brothers, Janice Brahney, Jennifer Weathered, Edd Hammill, and Trisha B. Atwood *

† These authors contributed equally to this work.

Correspondence to: [trisha.atwood@usu.edu](mailto:xxxxx@xxxx.xxx)

*Table. S1:* ***Endangered terrestrial tetrapod’s by percentage of land management type*
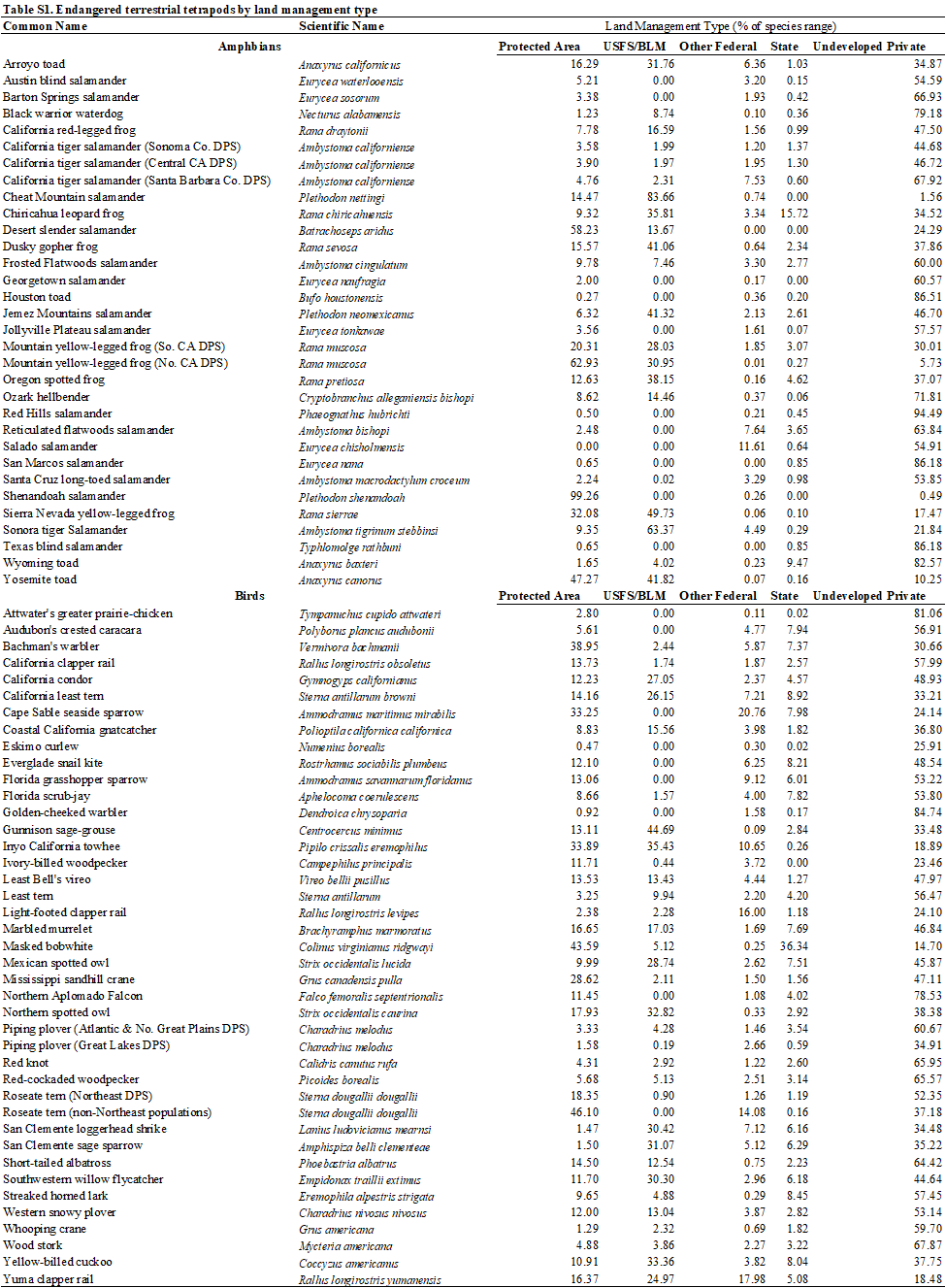
**

Continued on next page.


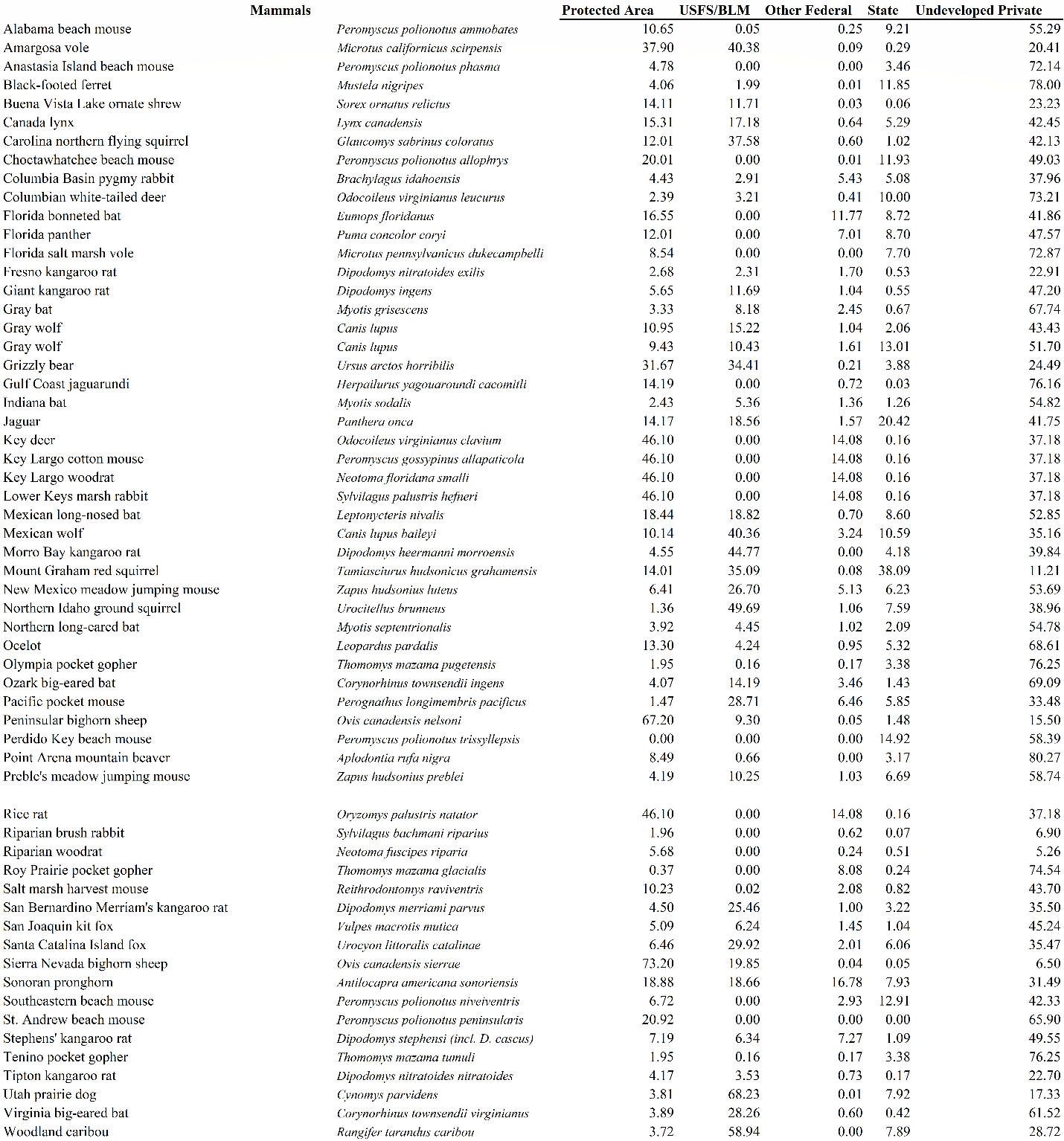
 ****

Red wolf *Canis rufus*  Populations considered experimental*.*

Continued on next page.

*Table. S2:* ***Endangered terrestrial tetrapod’s by percent of range in conservation easements***

Continued on next page.

Continued on next page.

Continued on next page.
